# Signal-On Fluorescence Biosensor for Highly Sensitive Detection of miRNA-21 Based on DNAzyme Assisted Double-Hairpin Molecular Beacon

**DOI:** 10.3390/bios12050276

**Published:** 2022-04-27

**Authors:** Chenxin Fang, Yuxing Yang, Shuhao Zou, Ping Ouyang, Yang Qing, Jialun Han, Haiyu Li, Zhencui Wang, Jie Du

**Affiliations:** College of Materials Science and Engineering, Hainan University, Haikou 570228, China; 19085204210012@hainanu.edu.cn (C.F.); 20085600210065@hainanu.edu.cn (Y.Y.); 20192113310016@hainanu.edu.cn (S.Z.); otping@hainanu.edu.cn (P.O.); 20080500210023@hainanu.edu.cn (Y.Q.); 18085204210013@hainanu.edu.cn (J.H.); 20080500110012@hainanu.edu.cn (H.L.); 20080500110014@hainanu.edu.cn (Z.W.)

**Keywords:** signal amplification, fluorescent biosensor, DNAzyme, miRNA detection, high sensitivity

## Abstract

Although miRNAs exist in small quantities in the human body, they are closely related to the abnormal expression of genes in diseases such as tumors. Therefore, sensitive detection of miRNAs is very important for the prevention and treatment of various tumors and major diseases. The purpose of this study is to develop a label-free sensing strategy based on the co-action of double-hairpin molecular beacons and deoxyribozymes (DNAzymes) for highly sensitive detection of miRNA-21. The target miRNA-21 promotes the assembly of DNAzyme with a complete catalytic core region. At the presence of Mg^2+^, DNAzyme cuts a substrate into short chains, which open the double hairpin molecular beacon, and then form G-quadruplexs at both ends, specifically binding more ThT to generate a amplified fluorescent signal. The cut substrate will be replaced by the uncut ones in the next stage, increasing the concentration of reactants, and thus further improving the fluorescence intensity. This DNAzyme assisted double hairpin molecular beacon has a certain degree of discrimination for substances with single base mismatches, and the detection limit of miRNA-21 is 0.13 pM, lower than that of the many other analysis. Further, this detection has good selectivity and sensitivity in serum. Therefore, this strategy provides a simple, fast and low-cost platform for the sensitive detection of miRNA-21, having potential applications in early cancer diagnosis.

## 1. Introduction

Nucleic acid is a class of biopolymer, which is an essential component of all known life forms and the most important substance in all biological molecules. Nucleic acids generally include deoxyribonucleic acid (DNA) and ribonucleic acid (RNA); the difference lies in the five-carbon sugars in the components [1]. DNA is abundant in organisms and stores most of the genetic material. DNA carries the genetic information necessary for the synthesis of RNA and proteins, and is an essential biological macromolecule for the development and normal operation of organisms. RNA is also responsible for regulating the genetic material in the organism, and its role is mainly to guide the synthesis of proteins. The joint action of DNA and RNA can play a role in regulating the expression of various genes, including the expression of various disease genes. Therefore, the detection of DNA and RNA is of great significance for the prediction and diagnosis of various diseases.

Among all kinds of RNA, there is a special kind of RNA, miRNA. miRNA is a kind of endogenous small molecule non-coding RNA with a length of about 20–24 nucleotides. Its content in the body is extremely low, but it has a variety of important regulatory roles in cells. A kind of miRNA can Regulates multiple genes, and there are also situations where multiple miRNAs regulate a gene [2]. The first miRNA was discovered in 1993 [3]; since then, the research on miRNA has gradually increased. Several studies have shown that abnormal expression of miRNA can lead to pathological changes in organisms, such as various types of cancer [4,5], various cardiovascular diseases [6], AIDS [7], diabetes [8], Parkinson’s disease [9], etc. Therefore, miRNAs are a kind of important biomarker. However, miRNAs have the problems of low abundance and many homologous miRNAs, and it has been a serious challenge to develop high-sensitivity and high-specificity miRNA detection strategies in the past few decades.

In my previous work, I proposed a fluorescence sensing strategy based on the combined combination of rolling circle amplification and DNAzyme to detect miRNA. The detection system uses miRNA as a promoter to trigger the rolling circle amplification reaction. The long-chain DNA produced by amplification can promote the formation of DNAzyme structure, promote the substrate cleavage reaction, and finally realize the separation of fluorophore and quencher group to generate fluorescence. The sensor has good selectivity and sensitivity.

Traditional detection methods for miRNA include northern blotting [10], microarrays [11] and reverse transcription PCR (RT-PCR) [12]. Among them, northern blotting and microarrays have a certain sensitivity and selectivity for detecting miRNA, but these methods are cumbersome in steps, complicated in operation, long in detection period and high in cost, and have certain limitations. RT-PCR, as a method for introducing fluorescent signal into the system to detect miRNA, has strong specificity and sensitivity. However, RT-PCR needs to strictly control the temperature, and there are false positive signals in the detection results. These factors limit this method. Application of detection methods. Therefore, it is necessary to find novel sensing strategies that can accurately detect miRNAs.

Emergence of functional nucleic acids provides a new approach for the development of novel sensing strategies [13,14,15]. Functional nucleic acids are a class of oligonucleotides with specific recognition functions. Such nucleic acids are widely used in the construction of various sensors, and the most widely used are nucleic acid aptamers. Nucleic acid aptamers, also known as chemical antibodies, have the advantages of no immune rejection, easy synthesis, good chemical stability, and can be stored at room temperature. Nucleic acid aptamers can specifically bind to various substances (proteins, nucleic acids, ions, glucose, etc.), and the DNA conformation changes after binding, resulting in specific changes [16,17,18]. DNA sensors constructed with nucleic acid aptamers have obvious advantages.

Another type of functional nucleic acid is Deoxyribozyme (DNAzyme), which not only has specific recognition function, but also has special catalytic properties. The concept of DNAzyme was first proposed in 1994 [19], Breaker et al. found that DNAzyme can cleavage RNA under the catalysis of Pb^2+^. After that, various ion-dependent DNAzymes were discovered, and more and more people introduced DNAzymes into the field of biosensors [20]. There are many kinds of DNAzymes, of which the most studied is the cleavage-type DNAzyme. There are two regions in the structure of the cleavage-type DNAzyme, the catalytic core region and the substrate-binding region, and the substrate–substrate-binding region can pass through alkali. Under the influence of catalytic ions, the DNAzyme is activated to undergo a substrate cleavage reaction [21]. Compared with proteases with similar catalytic functions, DNAzyme have many advantages: low synthesis cost, relatively high catalytic efficiency, low temperature requirements, and little change in catalytic activity after repeated denaturation and renaturation, excellent biological properties. Capacitance. Therefore, the introduction of DNAzymes into biosensors has a good prospect. Xiang et al. proposed a label-free DNA biosensor based on allosteric cleavage DNAzymes for the detection of miRNA and viral DNA, which has high sensitivity and specificity [22]. This experiment introduces a new type of DNAzyme, which includes not only the catalytic core area and substrate binding zone, but also an assembly promotion zone. The existence of the assembly promotion zone can improve the assembly efficiency of DNAzyme, and the sequence of the assembly promotion zone and the target detection object is complete. The presence of target detectors can help DNAzyme quickly assemble, thereby greatly improving detection efficiency, and ordinary cutting DNAzyme has limitations.

G-quadruplex is the secondary structure of a class of nucleic acids, and G-quadruplex is a higher-order structure formed by the folding of guanine (G)-rich DNA or RNA. G-quartet (G-quartet) is the structural unit of G-quadruplex. Four Gs are connected by Hoogsteen hydrogen bonds to form a ring plane, and two or more layers of tetrads form quadruplexes through π-π stacking [23]. The concept of G-quadruplex was formally proposed by Sen et al. in 1988 [24], and Mohanty et al. discovered for the first time that Thioflavin T can specifically bind to G-quadruplex, and revealed the mechanism of specific binding, resulting in an increase in fluorescence intensity [25]. After that, more and more researches on the G-quadruplex structure were conducted, and more and more people used the G-quadruplex structure in the field of biosensors. Zhang et al. designed a hairpin-type DNA-modified nanogel for the detection of platelet-derived growth factor (PDGF-BB). After the nanogel specifically bound to PDGF-BB, the fluorescence intensity increased, while the fluorescence signal of the system comes from ThT and G-quadruplex, and the sensor has good sensitivity and selectivity [26]. Chen et al. proposed a sensor for the detection of miRNA-21 based on the interaction of functionalized hairpin probes and isothermal amplification reactions. The fluorescence signal of the sensor is also derived from G-quadruplex and ThT [27]. This kind of label-free detection system is simple in operation, low in cost, and has strong sensitivity, which has certain advantages.

MB (Molecular Beacon) is a fluorescently labeled oligonucleotide chain. Molecular beacons generally consist of three parts: a ring region, a stem region, and a fluorescent group and a quenching group. When the molecular beacon is folded, the fluorescent group and the quenching group are close to each other, and the fluorescence is quenched. When the molecular beacon is opened, the two groups are far away from each other, and the fluorescence recovers [28]. Compared with the traditional detection system involving the structure of molecular beacon, the sensor involving the structure of unlabeled molecular beacon has advantages, such as low cost, high sensitivity, etc. Shahsavar et al. proposed a label-free DNA sensor based on molecular beacons for the detection of miRNA. The sensor has high sensitivity and specificity, and has better application prospects than ordinary labeled sensors [29]. Based on exponential isothermal amplification and two types of molecular beacons as the source of fluorescent signals, Liu et al. established a sensing strategy combining two signal amplification methods, which is fast, sensitive and has high specificity [30].

In this study, we propose a DNAzyme-based double-hairpin molecular beacon for the detection of miRNA-21 as a label-free sensor. The design idea of the sensor is as follows: miRNA-21 drives the two oligonucleotide chains in the detection system to form a DNAzyme with a catalytic core region and a substrate-binding region. When there are substrates and Mg^2+^ in the system, substrate cleavage occurs. Reaction, the cut DNA fragments are used in the second step reaction; the second step reaction uses the product of the first step cleavage reaction as a reactant, and a double hairpin probe is added to the system, the cleavage product can open the double hairpin probe and generate G-quadruplex and fluoresce under the action of ThT and K^+^. This sensing strategy does not involve enzymes and does not require labeling of fluorophores, which reduces the cost and does not require strict temperature control during the experiment.

## 2. Experimental

### 2.1. Materials and Methods

Tris-HCl buffer solution (1 mol L^−1^, pH 7.4), thioflavin T (ThT), and RNase inhibitor were purchased from Solarbio Life Sciences (China). Magnesium chloride (MgCl_2_) and potassium chloride (KCl) were purchased from Macklin Biochemical Co., Ltd. (Shanghai, China). Ultrapure water was purchased from Dongsheng Biotech Co., Ltd. (Guangzhou, China).

MiRNA, three kinds of oligonucleotides and double hairpin molecular beacon (Table 1) were purchased from Sangon Biotech (Shanghai, China). 

RF-6000 fluorescence spectrometer (Shimadzu, Japan) was used to measure the fluorescence signal value. In this experiment, ThT was used in this experiment as the fluorescein, which can specifically bind to the G-quadruplex to generate fluorescence signal value. The excitation and emission wavelengths of the fluorescein were 425 nm and 500 nm, respectively. 

### 2.2. Preparation of Reagents

Preparing Tris-HCl solution (20 mmol L^−1^ in water, pH 7.4), KCl solution (20 mmol L^−1^ in Tris-HCl solution, pH 7.4), and MgCl_2_ solution (10 mmol L^−1^ in Tris-HCl solution pH 7.4): DNAzyme1, DNAzyme2, substrate and double hairpin molecular beacon (HP2) were diluted with Tris-HCl solution (20 mmol L^−1^, pH 7.4) to 10 μmol L^−1^, 10 μmol L^−1^, 10 μmol L^−1^, and 10 μmol L^−1^. The miRNA was diluted to 10 μmol L^−1^ with DEPC-treated water. The product was stored in a refrigerator at 4 °C. Preparing other reagents, RNase inhibitor (40 U μL^−1^), and ThT solution (10 mmol L^−1^ in water): Put the RNase inhibitor in the lower layer of the refrigerator for frozen storage (−20 °C), and put the ThT solution in the refrigerator (4 °C).

### 2.3. Design Strategy of Sensor

**Figure 1:**
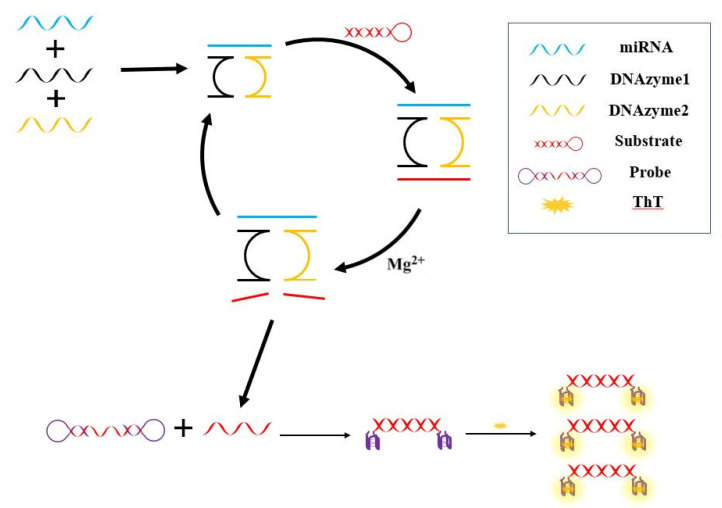
Schematic illustration of the fluorescence assay for the detection of miRNA-21 using DNAzyme and double hairpin molecular beacon.

Figure 1 showed the double hairpin probe strategy assisted by DNAzyme to detect miRNA-21. Firstly, when the target miRNA existed, it is bound with DNAzyme1 and DNAzyme2 through complementary base pairing, so miRNA-21 can effectively promote the assembly of these two DNAzymes. Initially, DNAzymes are inactive in the absence of magnesium ions and substrates. The substrate was designed as a hairpin structure, in order to avoid some nonspecific reactions in the system (for example, the straight chain substrate may directly open the double hairpin molecular beacon). At the presence of substrate and Mg^2+^, the activity of DNAzyme is activated, thus cutting the substrate. Then, the product after substrate cleavage combined with HP2, so that the hairpin structure at the two ends of the probe can be opened. Finally, in the presence of K^+^, G-quadruplex was formed, and ThT specifically combined with G-quadruplex to produce fluorescence. 

In the first stage of the reaction, the DNAzyme designed in this experiment continuously cuts the substrate, and the substance cleaved from the substrate can be used as the promoter of the next reaction, which can trigger the double hairpin DNA reaction. In the second stage of the reaction, the cleaved substrate can open the double hairpin structure, and the double hairpin molecular beacon has G-rich sequences at both ends. One molecular beacon has two G-quadruplex structures, which bind more fluorescein, and thus further amplifying the fluorescence signal. 

### 2.4. DNAzyme Assembly Reaction

Preparation 15 μL solution contains 1 μL target miRNA (the final concentration selected depends on the optimization result), 2 μL 8 nmol L^−1^ DNAzyme1, 2 μL 8 nmol L^−1^ DNAzyme2, and 20 U RNase inhibitor. The above solution was shaken evenly, reacted at 65 °C for 5 min, and then reacted at 37 °C for 2 h.

### 2.5. Substrate Cutting Reaction

The substrate was annealed at 95 °C and slowly cooled to room temperature and maintained at room temperature for at least 1 h to ensure that the substrate can form an ideal hairpin structure. Added 2 μL 8 nmol L^−1^ of substrate and 1 μL 4 μmol L^−1^ Mg^2+^, and reacted at 37 °C for 4 h to carry out the substrate cleavage reaction.

### 2.6. Binding of ThT to G-Quadruplex

The HP2 underwent the same annealing treatment as the substrate, ensuring the formation of the double-hairpin structure. First, 59 μL of 20 mmolL^−1^ Tris-HCl solution containing 2 μL 8 nmol L^−1^ HP2 and 1 μL 8 nmol L^−1^ K^+^ were reacted at 37 °C for 2.5 h. Finally, 1 μL 2 μmol L^−1^ ThT was added to the system and the reaction was carried out at 37 °C for 0.5 h, finally the reaction product was placed in a refrigerator for fluorescence testing.

## 3. Results and Discussion

### 3.1. Feasibility Analysis

In this experiment, the assembly of DNAzymes was promoted by the presence of target miRNA. In the presence of different concentrations of target miRNA, the number of DNAzymes formed was also different, the cutting sites of substrates will be different, and the open double hairpin molecular beacon will be different, which will eventually lead to different fluorescence intensity. The higher the concentration of target miRNA, the higher the efficiency of formation of DNAzymes, the more cleavage sites formed, and the more substrates that can be used for the next step. Finally, the more G-quadruplexes formed, and the higher the fluorescence intensity. 

**Figure 2:**
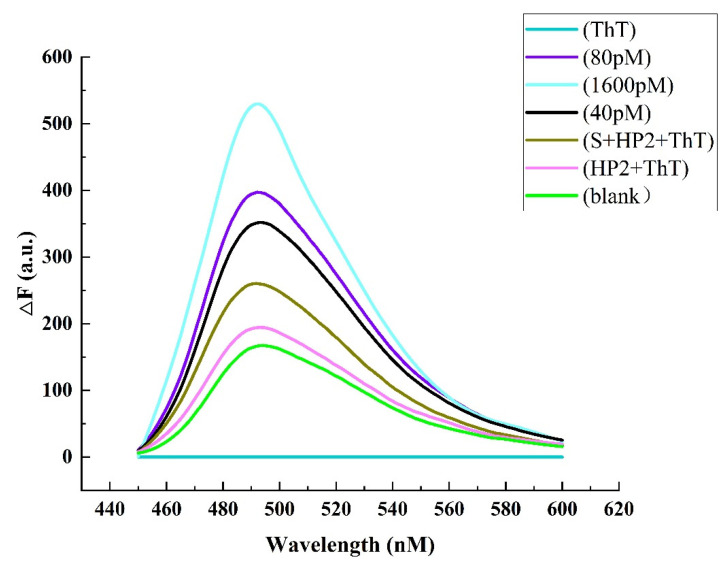
Fluorescence spectrum in the absence of sensing system ingredients.

Further, six groups of experiments were selected for feasibility analysis, namely with only ThT system; HP2 and ThT system; S, HP2 and ThT system; the blank system (the system without target detector); 40 pM (the target concentration is 40 pmol L^−1^); 80 pM (the target concentration is 80 pmol L^−1^) and 1.6 nM (the target concentration is 1.6 nmol L^−1^). As shown in Figure 2, the fluorescence intensity of only ThT, HP2 and S + HP2 systems is significantly lower, indicating that the fluorescence intensity of the system will be reduced without any substance. The blank system, 40 pM system, 80 pM system and 1,6 nM system show the trend that the higher the concentration of the target detector, the higher the fluorescence intensity, indicating that the target detector miRNA-21 participated in the above reaction. Therefore, the feasibility of this experiment can be verified from Figure 2. 

### 3.2. DNAzyme1 and DNAzyme2 Concentration Optimization

In this experiment, DNAzyme1 and DNAzyme2 formed the complete structure of cleaved DNAzymes, which includes the catalytic core region, substrate binding region and promoting assembly region. In the presence of the target, the combination of the assembly region is promoted through base complementary pairing, and thus DNAzyme1 and DNAzyme2 can be close to each other, resulting in the formation of the catalytic core region and the efficiency of cutting the substrate. Therefore, the catalytic core region formed by the combination of DNAzyme1 and DNAzyme2 is an important factor affecting the experimental results.

**Figure 3:**
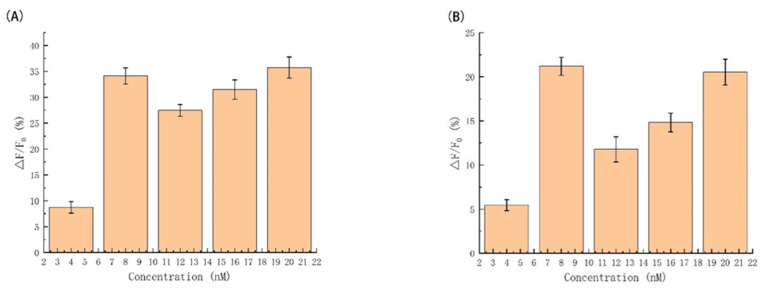
Fluorescence intensity (∆F) in the presence of (**A**) different DNAzyme1; and (**B**) DNAzyme2 dosages.

Figure 3A shows the relationship between the concentration of DNAzyme1 and fluorescence intensity. With the gradual increasing concentration of DNAzyme1 from 0 to 16 nmol L^−1^, the fluorescence signal value firstly increases and then decreases. When 8 nmol L^−1^ reaches the maximum value of fluorescence signal ratio, so 8 nmol L^−1^ was chosen as the concentration of DNAzyme1 for next subsequent optimization experiments. 

In principle, the function of DNAzyme2 is the same as that of DNAzyme1, which together forms the catalytic core region. At the presence of Mg^2+^, the catalytic core region is activated to cut the substrate. Figure 3B shows the relationship between the concentration of DNAzyme2 and fluorescence intensity. With the concentration of DNAzyme2 from 0 to 16 nmol L^−1^, the fluorescence signal value also initially increases and then decreases, reaching the maximum value of fluorescence intensity at 8 nmol L^−1^. Therefore, 8 nmol L^−1^ as the concentration of DNAzyme2 finally was chosen for subsequent optimization experiments and selectivity analysis. 

### 3.3. Optimization of Substrate Concentration

**Figure 4:**
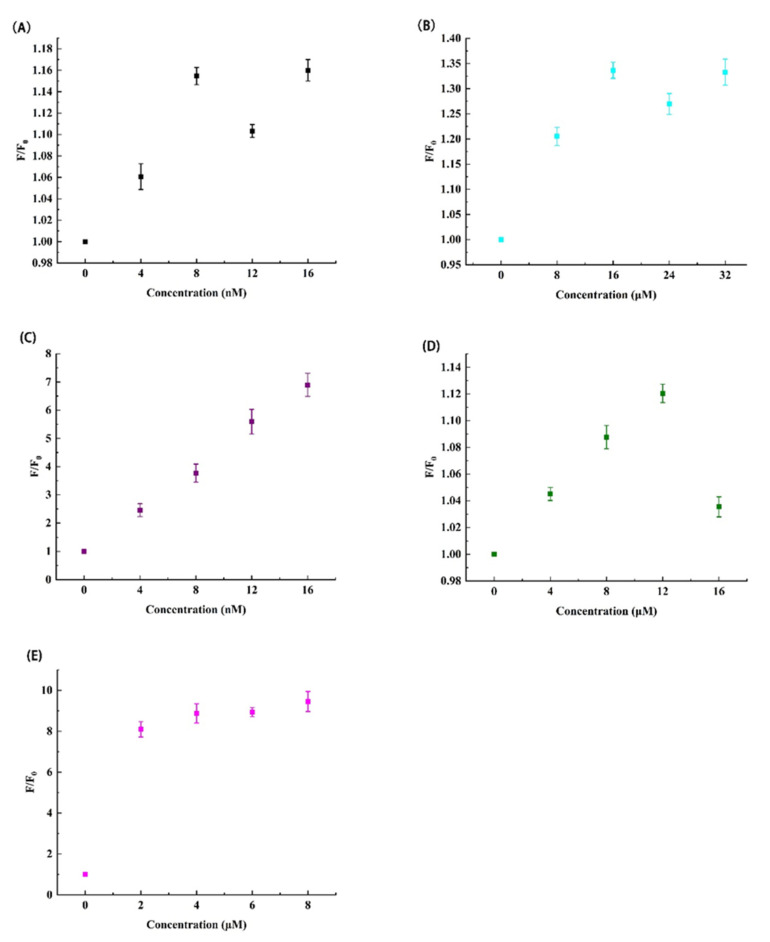
Fluorescence intensity in the presence of different dosages of each factor (**A**) Substrate; (**B**) Mg^2+^; (**C**) HP2; (**D**) K^+^; and (**E**) ThT.

The substrate of the general experiment was designed as a DNA with a straight-chain structure, and the substrate of this experiment was designed as a hairpin structure to reduce systematic errors. There is a ribonucleotide in the middle of the substrate, which can be successfully cut by DNAzyme. Under normal circumstances, the substrate cannot be opened, and the substrate can only be opened when the perfectly complementary sequence is present. The designed sequence of the substrate binding region in this experiment is exactly complementary to the substrate sequence, which minimizes the uncertainty of the experiment. After the substrate is bound to the substrate binding region, Mg^2+^ promotes DNAzyme to carry out the substrate cleavage reaction. The more substrates are cleaved, the more double hairpins are opened. Therefore, theoretically, the relative fluorescence intensity increases with increasing substrates. This estimation was confirmed in Figure 4A. However, note that the relative fluorescence intensity increases slowly with the concentration from 0 to 8 nmol L^−1^, while remains unchanged more than 8 nmol L^−1^. From the cost factor point of view, 8 nmol L^−1^ was selected as the optimal concentration of the substrate for subsequent experiments. 

### 3.4. Optimization of Mg^2+^ Concentration 

When DNAzyme structure forms, although it can match with the substrate through base complementary pairing, the substrate cutting reaction cannot be carried out due to the lack of Mg^2+^. In general, the catalytic activity of DNAzyme depends on the concentration of Mg^2+^. Therefore, the existence of Mg^2+^ determines the cutting efficiency of DNAzyme and ultimately affects the fluorescence signal value. 

As shown in Figure 4B, when the concentration of Mg^2+^ increases from 0 to 32 μmol L^−1^, the overall trend of its relative fluorescence intensity increases gradually and then becomes stable. However, when the concentration of Mg^2+^ increases from 16 to 24 μmol L^−1^, the relative fluorescence intensity shows a downward trend. In addition, when the Mg^2+^ concentration is 32 μmol L^−1^, the relative fluorescence intensity increases slightly, but almost the same as that of Mg^2+^ concentration of 16 μmol L^−1^. Therefore, from the perspective of economy and obvious results, 16 μmol L^−1^ was chosen as the Mg^2+^ concentration for subsequent optimization experiment and specificity analysis experiment.

### 3.5. Optimization of HP2 Concentration 

Double hairpin Molecular beacon was an important part of generating fluorescence intensity. After DNAzyme was activated, it can efficiently cut the substrate. Part of the substrate cut down can open the HP2. The more double hairpin structures were opened, the more G-quadruplex sequences would be generated. G-quadruplex sequences would form G-quadruplex structure and generate fluorescence intensity at the presence of K^+^ and ThT. Therefore, theoretically, the more the double hairpin probe was opened, the more the G-quadruplex structure and the higher the fluorescence intensity. Figure 4C shows the relationship between relative fluorescence intensity and probe concentration. As predicted in theory, the relative fluorescence intensity increased with increasing probe’s concentration (from 0 to 16 nmol L^−1^). From the perspective of experimental resulted and economic benefits, 8 nmol L^−1^ was selected as the probe concentration for subsequent optimization of other factors.

### 3.6. Optimization of K^+^ Concentration 

In this experiment, K^+^ played a role in promoting the assembly of sequences with G-quadruplex bases into G-quadruplex DNA structures. When the double hairpin structure was opened by the cut substrate, the G-quadruplex base sequence at the end cannot form a G-quadruplex structure, which needed the stimulation of external factors to form a specific structure. G-quadruplex sequence was a specific base sequence rich in G. metal ions can induce G-rich nucleic acid aptamer to form G-quadruplex and maintain the stability of the structure. Therefore, K^+^ can induce and stabilize the formation of G-quadruplex structure. 

As shown in Figure 4D, with the increasing of K^+^ concentration, the relative fluorescence intensity increased and then decreased. The overall situation was consistent with the theoretical prediction. Less than 12 μmol L^−1^, the relative fluorescence intensity was lower than that at 12 μmol L^−1^, while more than 12 μmol L^−1^, the relative fluorescence intensity ratio decreased. Therefore, 12 μmol L^−1^ was selected as the optimal concentration of K^+^ for next study. 

### 3.7. Optimization of ThT Concentration 

DNA with a specific base sequence (generally containing multiple G) can form a G-quadruplex rich structure composed of special hydrogen bonds under the action of fluorescent dye Thioflavin T, and finally increase the fluorescence intensity of the system. Therefore, in this experiment, not only K^+^ can induce and stabilize G-quadruplex, but ThT fluorescein would also induce the end sequence of double hairpin structure to form G-quadruplex structure. In this experiment, ThT can specifically bind to G-quadruplex structure, so as to significantly increase the fluorescence intensity. Therefore, the more ThT, the higher the fluorescence intensity in theory. 

It is obvious from Figure 4E that with the concentration of ThT from 0 to 8 μmol L^−1^, its relative fluorescence intensity increased slowly. However, the increase was more obvious from 0 to 4 μmol L^−1^, and the relative fluorescence intensity of 4 μmol L^−1^ to 8 μmol L^−1^ did not change much. It was speculated that the double hairpin structure has limited G-quadruplex binding sites, and a fixed number of sites can only bind a fixed number of ThT, resulting in little change in relative fluorescence intensity. Therefore, 4 μmol L^−1^ was finally selected as the optimal concentration of ThT for subsequent concentration gradient analysis. 

### 3.8. Sensitivity Analysis of MiRNA-21 

The purpose of this study is to design a DNAzyme-based biosensor to sensitively and rapidly detect target miRNA. The optimized experimental conditions were DNAzyme1 (8 nmol L^−1^), DNAzyme2 (8 nmol L^−1^), substrate (8 nmol L^−1^), Mg^2+^ (16 μmol L^−1^), HP2 (8 nmol L^−1^), K^+^ (12 μmol L^−1^), and ThT (2 μmol L^−1^). In these cases, the fluorescence signal value can reach the maximum. According to the standard steps of the experiment, the detection system was used to detect different concentrations of target miRNA-21, so as to explore the sensitivity of target miRNA and obtain the relationship between miRNA-21 and fluorescence intensity at different concentrations. Each group of experiments was repeated three times. 

**Figure 5:**
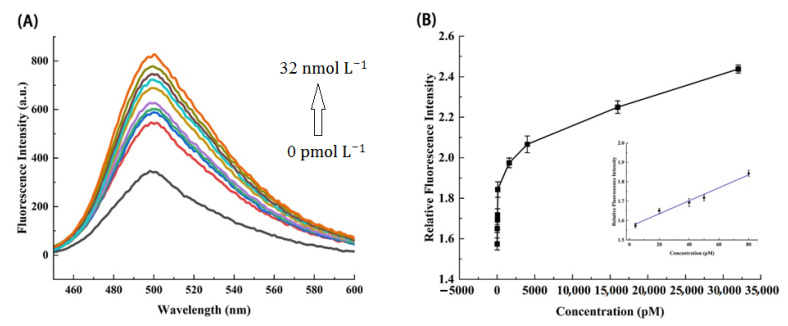
(**A**) Fluorescence spectra in the presence of different concentrations of miRNA-21 (in buffer). (**B**) Plot of relative fluorescence intensity versus miRNA-21 concentration at 500 nm emission wavelength. (The inset shows the relationship between target concentration and relative fluorescence intensity in the concentration range from 0 to 100 pM). The error bars represent the standard deviation of three repeat experiments.

As shown in Figure 5A, within the range of 0 pmol L^−1^ to 32 nmol L^−1^, the fluorescence intensity increases gradually with the gradual increasing concentration of miRNA-21. When the concentration of the target detector is as low as 4 pmol L^−1^, there is still a large difference between the fluorescence intensity at this concentration and that without the target, indicating that this detection strategy processes a high sensitivity, even with the pM level concentration of miRNA-21. Figure 5B shows the relationship between the relative fluorescence intensity at 500 nm and the concentration from 4 pmol L^−1^ to 32 nmol L^−1^. After simple fitting, the curve has a good linear relationship between 4 pmol L^−1^ and 80 pmol L^−1^ (R^2^ = 0.97463). The limit of detection (LOD) was based on three times the standard deviation (3σ) of the blank signal. It was calculated that the detection limit was 0.13 pM, estimated to be three times of the blank (without miRNA-21) standard deviation divided by the slope (3σ/S). The limit of quantification is 10* Blank standard deviation/slope (10σ/S) and the LOQ was 0.433 pM. The signal to noise ratio (SNR), which means the ratio of signal value to background signal, can reach more than 2, indicating that the system has a lower background signal. The results shows that this sensing system can detect the target substance more accurately. 

### 3.9. Specificity Analysis of MiRNA-21

Further, under the same optimized conditions, the signal values obtained from the detection of other homologous miRNAs similar to miRNA-21 were investigated. Theoretically, if there is mismatched miRNA or non-target miRNA, the efficiency of the miRNA driving the next reaction decreases, and eventually leads to a significant decrease in fluorescence intensity. In order to make the experiment more convincing, a variety of miRNAs were selected for specific detection (miRNA-205, miRNA-141, miRNA-210, miRNA-221, let-7a, miRNA-16, mut-miRNA-21). Among them, except that the concentration of the detection substance in miRNA-21 group was 40 pmol L^−1^, the concentration of the detection substance in other groups was 0.4 nmol L^−1^.

**Figure 6:**
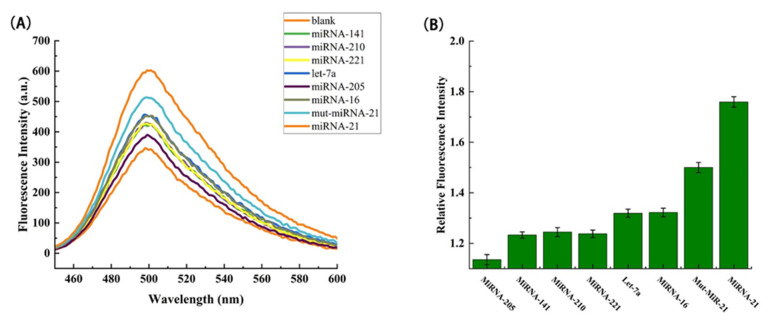
(**A**) Fluorescence spectrum of the relationship between different types of miRNAs and fluorescence intensity. (**B**) The difference in relative fluorescence intensity at the emission wavelength of 500 nm and different miRNA species, where error bars represent the standard deviation of three replicate experiments.

Figure 6A showed the fluorescence spectra of various detection substances. It can be clearly seen from the figure that the fluorescence intensity of the experimental group of multiple miRNA-21 homologous miRNAs was lower than that of the control group of miRNA-21 with a concentration of one order of magnitude lower. As can be seen from Figure 6B, the relative fluorescence intensity of the control group is much higher. Compared with the single base mismatch experimental group, it also had a certain degree of discrimination. In general, this method had a good ability to distinguish the single base mutant miRNA from other homologous miRNAs, and had good specificity and selectivity. 

### 3.10. Actual Sample Detection of miRNA-21

In order to further explore the application, this experiment measured the actual samples in serum, and detected the stability and sustainability of the sensing system in the complex biological environment by adding different concentrations of target to the reaction system with serum. In this experiment, four different concentrations of miRNA-21 were added to the diluted serum samples, and the substance to be tested with concentration A was added. The concentration of the substance to be tested was determined by the regression curve as B, and the recovery rate was (A/B) × 100%.

Table 2 shows that the recovery of the detection method for the target detection object (miRNA-21) is 107.5%, 97.1% and 90.6%, respectively, and the relative standard deviations of the recovery is 6.7%, 5.1% and 8.2%, respectively. The recovery basically meets the basic requirements of the experiment. The experimental results show that the detection method can be more accurate in the complex biological environment, thus having great potential prospects in the future application stage. 

## 4. Conclusions

In this paper, a label-free, low-cost DNA biosensor was constructed using DNAzyme and double hairpin structure. The sensor is driven by the target miRNA-21 to facilitate the assembly of two types of DNAzymes to form DNAzymes with complete structures. After the hairpin substrate and Mg^2+^ are added to the system, the cleavage DNAzyme is activated and the substrate cleavage reaction occurs. The fragment of the cleaved substrate can open the double hairpin structure, and the end of the opened double hairpin structure can form a G-quadruplex in the presence of ThT and K^+^, and the G-quadruplex can bind to ThT. As a result, the fluorescence intensity is greatly increased. The experimental results show that the detection method has good selectivity and can be used for quantitative analysis. In addition, the detection method can also be applied in a relatively complex serum environment. Therefore, the experimental method has strong feasibility and has a great prospect to be widely used in disease prevention and treatment.

## Figures and Tables

**Table 1:** Sequences of the oligonucleotides used in this study.

Name	Sequence (from 5′ to 3′)
DNAzyme1	GGG TGT TGA TGGG AGC GAT CTT CTG ATA AGC TA
DNAzyme2	TCA ACA TCA GT AAG CAC CC ATG TCC CAT CAA CAC CC
Substrate	GGG TGT TGA TGGG T/rA/G CCC ATC AAC ACCC
HP2	GGG TAG GGC GGG TTG GGC CCC ATC AACA CCC CGG GTT GGG CGG GAT GGG
MiRNA-21	UAG CUU AUC AGA CUG AUG UUG A
MiRNA-16	UAG CAG CAC GUA AAU AUU GGC G
Mut-MiRNA-21	UAG CUU AAC AGA CUG AUG UUG A
MiRNA-141	UAA CAC UGU CUG GUA AAG AUG G
MiRNA-205	UCC UUC AUU CCA CCG GAG UCU GU
MiRNA-221	AGC UAC AUU GUC UGC UGG GUU UC
MiRNA-210	CUG UGC GUG UGA CAG CGG CUG A
Let-7a	UGA GGU AGU AGG UUG UAU AGU U

**Table 2:** Recovery results of miRNA-21 in serum samples.

Added (pM)	Found	Recovery (%)	RSD (%, *n* = 3)
20	18.6	107.5	6.7
40	41.2	97.1	5.1
60	66.2	90.6	8.2

## Data Availability

Not applicable.
